# Comparative Chromosome Painting in Six Species of *Oligoryzomys* (Rodentia, Sigmodontinae) and the Karyotype Evolution of the Genus

**DOI:** 10.1371/journal.pone.0117579

**Published:** 2015-02-06

**Authors:** Camilla Bruno Di-Nizo, Karen Ventura, Malcolm Andrew Ferguson-Smith, Patricia Caroline Mary O’Brien, Yatiyo Yonenaga-Yassuda, Maria José de J. Silva

**Affiliations:** 1 Laboratório de Ecologia e Evolução, Instituto Butantan, São Paulo, São Paulo, Brazil; 2 Departamento de Genética e Biologia Evolutiva, Instituto de Biociências, Universidade de São Paulo, São Paulo, São Paulo, Brazil; 3 Cambridge Resource Centre for Comparative Genomics, Department of Veterinary Medicine, University of Cambridge, Cambridge, United Kingdom; 4 Instituto de Recursos Naturais—Universidade Federal de Itajubá, Itajubá, Minas Gerais, Brazil; University of Florence, ITALY

## Abstract

*Oligoryzomys* belongs to the tribe Oryzomyini, and contains about 22 species. Diploid numbers range from 2n = 44 in *Oligoryzomys* sp. 2 to 2n = 72 in *O. utiaritensis* and phylogenetic relationships are not well defined. The high morphological convergence leads to misidentification of taxonomic entities and the species are often identified by chromosomal characters. Until now, the genus has been studied only by classical cytogenetic approaches. To understand the chromosomal evolution of *Oligoryzomys*, we developed chromosome probes from a female of *Oligoryzomys moojeni* (OMO) with 2n = 70 and hybridized to other five *Oligoryzomys* species. The probes painted 31 segments on *O. fornesi* (OFO) with 2n = 62; 32 segments on *O. microtis* (OMI), 2n = 64; 33 segments on *O. nigripes* (ONI), 2n = 62 and on *O. rupestris* (ORU), 2n = 46; and 34 on *Oligoryzomys* sp. 2 (OSP), 2n = 44. OMO probes 4 and 5 showed a syntenic association in *O. fornesi*, *O. microtis* and *O. nigripes* and were also presented in the same pair, although disrupted, in *O. rupestris* and *Oligoryzomys* sp. 2. Concerning *O. rupestris* and *Oligoryzomys* sp. 2, species with the lowest diploid numbers of the genus, a total of 8 probes hybridized to 11 segments on the largest pair of ORU 1 and 9 probes hybridized to 12 segments on OSP 1. Also, OMO 6 painted three segments in ORU, corresponding to the proximal segment of ORU 2q, and the whole of ORU 19 and 20. In OSP, the segment corresponding to ORU 20 was homologous to OSP 1p. OMO X showed signals of hybridization in both X and Y chromosomes. Extensive chromosomal rearrangements, that could not be detected by classical cytogenetic techniques, such as pericentric inversions or repositioning of centromeres, Robertsonian rearrangements and tandem fusions/fissions, as well as gain/activation or loss/inactivation of centromeres and telomeric sequences have driven the huge genome reshuffling in these closely related species.

## Introduction

Rodents of the subfamily Sigmodontinae represent one of the most complex groups of New World mammals [1]. Traditionally, Sigmodontinae have been divided into tribes [2,3], and nowadays nine tribes are recognized, in addition to several *incertae sedis* genera that could not be grouped in any tribe [1,3].

Oryzomyini is the most diverse Sigmodontinae tribe and this is reflected in morphological, ecological and chromosomal variations which lead to taxonomic controversies [4,5]. One of the most complex genus in terms of taxonomic issues and number of species is *Oligoryzomys*.

*Oligoryzomys* is a speciose genus widely distributed from Mexico to Argentina, [6]. Phylogenetic analyses using morphology, allozymes and DNA sequences agree on the monophyly of the genus although the hierarchical relationships among species are not well established [7–11]. Recently, Agrellos et al. [12] based on nuclear (intron 7 of the beta-fibrinogen) and mitochondrial (cytocrome *b*) gene sequences, found *O*. *microtis to be the sister group of all other Oligoryzomys species while O*. *moojeni and O*. *utiaritensis clustered together in the more derived clade*.

The high morphological similarity generates difficulties in establishing the real number of species [12,13]. Heretofore, 22 species have been recognized but taxonomic problems still persist. Because of this, most species of the genus have been diagnosed by diploid (2n) and fundamental number (FN), instead of morphology [11,14].

Currently, cytogenetic studies in this group show great variation in diploid number, from 2n = 44 in *Oligoryzomys* sp. 2 to 2n = 72 in *O*. *utiaritensis* [12,15,16]. These studies reveal several chromosomal rearrangements including pericentric inversions, sex chromosomes polymorphisms and the presence of supernumerary chromosomes (Bs) [17,18].

Silva and Yonenaga-Yassuda [15] described the karyotypes of *O*. *rupestris* (2n = 46, FN = 52) and *Oligoryzomys* sp. 2 (2n = 44, FN = 52) and suggested that centric fusion was responsible for differences in diploid number between these species. In addition, when compared with other *Oligoryzomys* species, *O*. *rupestris* and *Oligoryzomys* sp. 2 present very distinctive karyotypes, with macro- and micro-chromosomes [15,16]. In contrast, the majority *Oligoryzomys* species possess chromosomes with a gradual variation in size and mostly with acrocentric morphology.

Heretofore, cytogenetic studies in *Oligoryzomys* used only banding patterns and fluorescent in situ hybridization (FISH) with telomeric probes [15,16]. However, cytogenetic comparison using banding patterns may not be informative enough when it comes to species with highly divergent genomes, such as *Oligoryzomys* [19,20].

This work brings to light a more refined perspective on karyotype evolution of *Oligoryzomys* as it is the first study using chromosome painting with species-specific probes in such a large number of species. The aim of this work is to investigate chromosome homologies among *Oligoryzomys* species and infer the rearrangements that have occurred during karyotype evolution, based on a published molecular phylogeny of the genus [12].

## Material and Methods

### Sample

The sample comprises seven species of *Oligoryzomys* from different localities of Brazil (Table 1): *O*. *flavescens* (OFL, 2n = 64+2B), *O*. *fornesi* (OFO, 2n = 62), *O*. *microtis* (OMI, 2n = 64), *O*. *moojeni* (OMO, 2n = 70), *O*. *nigripes* (ONI, 2n = 62), *O*. *rupestris* (ORU, 2n = 46) and *Oligoryzomys* sp. 2 (OSP, 2n = 44) [15]. Animals were euthanized according to the protocol of the “Animal experimentation ethics” [21]. The experiments were conducted according to the Committee on the Ethics of Animal Experiments of the Instituto Butantan (Comissão de ética no uso de animais do Instituto Butantan—permit number: 242/05). Skins and skulls were deposited at the Museu de Zoologia da Universidade de São Paulo (MZUSP). Spread metaphases of OFO, OMI, OMO, ONI, ORU and OSP were obtained from cell cultures, and in the case of OFL, from bone marrow.

**Table 1:** Species, diploid (2n), fundamental number (FN), sex, and collection localities of *Oligoryzomys* from Brazil.

Specimen number	Species	2n	FN	Sex	Locality	Geographic coordinate
ROD 21	*O. flavescens*	64+2B	66	M	Iperó, SP	23°25’S 47°35’W
CIT 1477	*O. fornesi*	62	64	M	Peixe, TO	12°01’S 48°32’W
CIT 696	*O. microtis*	64	64	M	Aripuanã, MT	10°10’S 59°27’W
CIT 2040	*O. moojeni*	70	72	F	Minaçu, GO	13°55’S 48°22’W
BIO 797	*O. nigripes*	62	81	M	Fazenda Intervales, SP	24°12’S 48°30’W
BIO 899*	*O. rupestris*	46	52	F	Pico das Almas, BA	13°33’S 41°56’W
BIO 813*	*Oligoryzomys* sp. 2	44	52	M	Serra do Cipó, MG	19°18’S 43°35’W

Brazilian states: SP = São Paulo; TO = Tocantins; MT = Mato Grosso; GO = Goiás; BA = Bahia; MG = Minas Gerais.

M = male; F = female.

*Samples firstly studied by Silva and Yonenaga-Yassuda [15].

### Generation of *Oligoryzomys moojeni* (OMO) chromosome paints

Specific painting probes were generated from fibroblast cultures of a female of *O*. *moojeni*, with 2n = 70 and heteromorphic X chromosomes, in the Cambridge Resource Centre for Comparative Genomics, Department of Veterinary Medicine, University of Cambridge, UK. The whole chromosome probes were made by degenerate oligonucleotide-primed polymerase chain reaction (DOP-PCR) as previously described [22,23]. Briefly, the chromosomes were prepared as described and stained with Hoechst 33258 (2 μg/ml) and Chromomycin A3 (40 μg/ml) in the presence of magnesium sulfate (2.5 mmol/l) for 2 h. Sodium sulfite (25 mmol/l) and sodium citrate (10 mmol/l) were added 15 min prior to flow sorting. Chromosome sorting was performed using a dual-laser cell sorter (MoFlo; Beckman Coulter). Approximately 400 chromosomes were sorted from each peak in the flow karyotypes directly into PCR tubes containing 30 μl of distilled water. Each sample was amplified by DOP-PCR using the primer 6MW [22]. Primary PCR products were labeled with biotin-16-dUTP (Boehringer Mannheim) or fluorescein isothiocyanate (FITC)-12-dUTP (Amersham) by taking 1 μl of product to a second round of DOP-PCR using the same primer. The biotin probes were detected with avidin-Cy3 or avidin-FITC.

### *In situ* hybridizations

In situ hybridization of *O*. *moojeni* painting probes was performed according to Yang et al. [23]. About 12 μl of hybridization buffer (50% deionized formamide, 10% dextran sulfate, 20x saline sodium citrate—SSC, 0.5 M phosphate buffer, pH 7.3) and 1 μl of labeled PCR product were denatured at 37° C for 30 min, dropped onto slides that were previously denatured in 70% formamide/ 2xSSC at 65° C for 2–3 min and mounted with coverslip. Cross-species hybridization was performed for 48–72 h at 37° C. Post-hybridization washes included 2×5 min incubations in 50% formamide/2x SSC at 42° C followed by 2×5 min incubations in 2x SSC and 4x T (100 ml 20x SSC, 400 ml H2O, 250 μl Triton X-100 Sigma-Aldrich). Slides were counterstained with 4’, 6-diamidino-2-phenylindole (DAPI) in the antifade Vectashield and analyzed in Zeiss Axiophot fluorescence microscope. On the hybridized metaphases, the chromosomes were identified by 4’,6-diamidino-2-phenylindole (DAPI) staining.

## Results

### Flow sorting of *O*. *moojeni* (2n = 70, XX)

**Fig 1:**
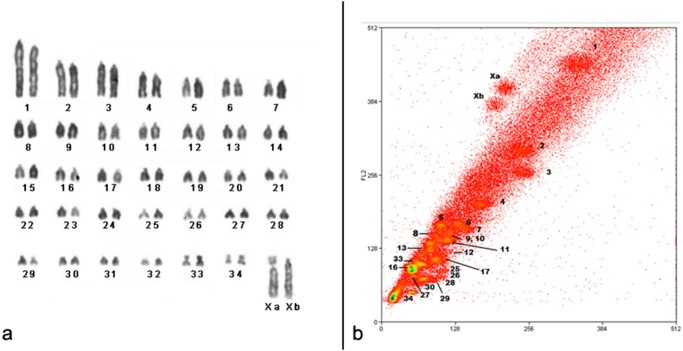
Karyotype and characterization of OMO probes. **(a)** Conventional stained karyotype of *Oligoryzomys moojeni* (2n = 70, FN = 72, female), with heteromorphic X, sample from which specific probes were sorted. (**b)** Flow karyotype of *O*. *moojeni* with the peaks that were identified. Chromosomes were sorted for DNA content and AT to CG base pair ratios after Hoechst 33258 (vertical axis) and chromomycin-A3 (horizontal axis) staining.

The karyotype of the female used for sorting had 32 acrocentric pairs (pair 1 is clearly the largest of the complement), two small biarmed pairs (33 and 34) and heteromorphic sex chromosomes: submetacentric (Xa) and subtelocentric (Xb) (Fig. 1a). The flow karyotype yielded 30 peaks in which only 23 hybridized to single pairs. Four peaks painted two pairs (one of them could be identified after double color hybridization) and three peaks painted three or four pairs. From that, 24 out of the 30 peaks could be identified (Fig. 1b) by fluorescent in situ hybridization to DAPI-banded metaphases (OMO Xa, Xb, 1–8, 9/10, 11–13, 16, 17, 25–30, 33, and 34). The double peak identified corresponded to pairs OMO 9/10. X chromosomes were separated into different peaks due to differences in size (detected after measurements) and in the amount of constitutive heterochromatin located in the short arm of both Xa and Xb, although Xa has a larger C+ block (data not shown). The remaining peaks could not be identified since they are composed of more than one pair and probably they overlap with other identified peaks; therefore they are not shown on Fig. 1b.

**Fig 2:**
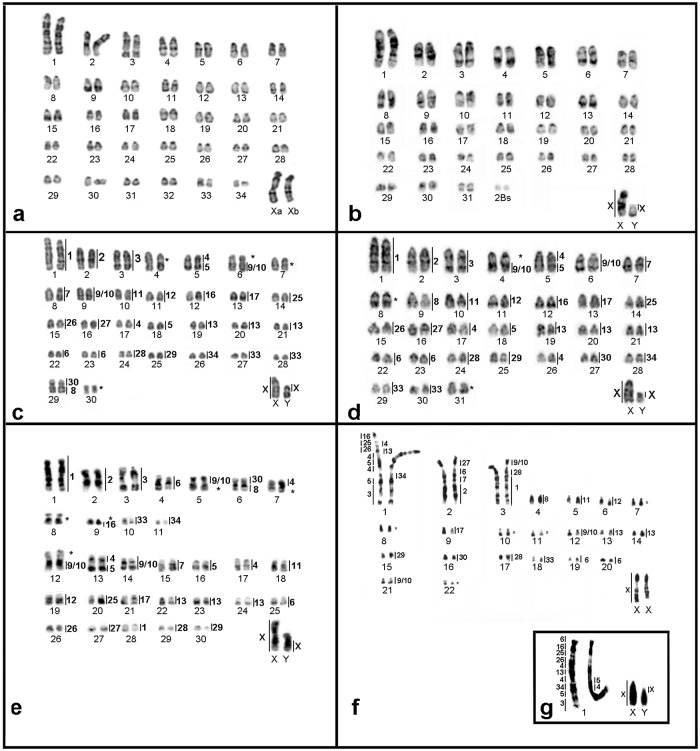
G-banded karyotypes of *Oligoryzomys* species studied in this work indicating the hybridization signals of OMO probes beside the chromosomes. (**a)** *O*. *moojeni* (OMO 2n = 70, FN = 72, female); (**b)** *O*. *flavescens* (OFL 2n = 64+2Bs, FN = 66, male); **(c)** *O*. *fornesi* (OFO 2n = 62, FN = 64, male); (**d)** *O*. *microtis* (OMI 2n = 64, FN = 64, male); (**e)** *O*. *nigripes* (ONI 2n = 62, FN = 81, male); (**f)** *O*. *rupestris* (ORU 2n = 46, FN = 52, female). (**g**) Pair 1 and sex chromosomes of *Oligoryzomys* sp. 2 (OSP 2n = 44, FN = 52, male). * Regions not hybridized by any OMO probes.

**Fig 3:**
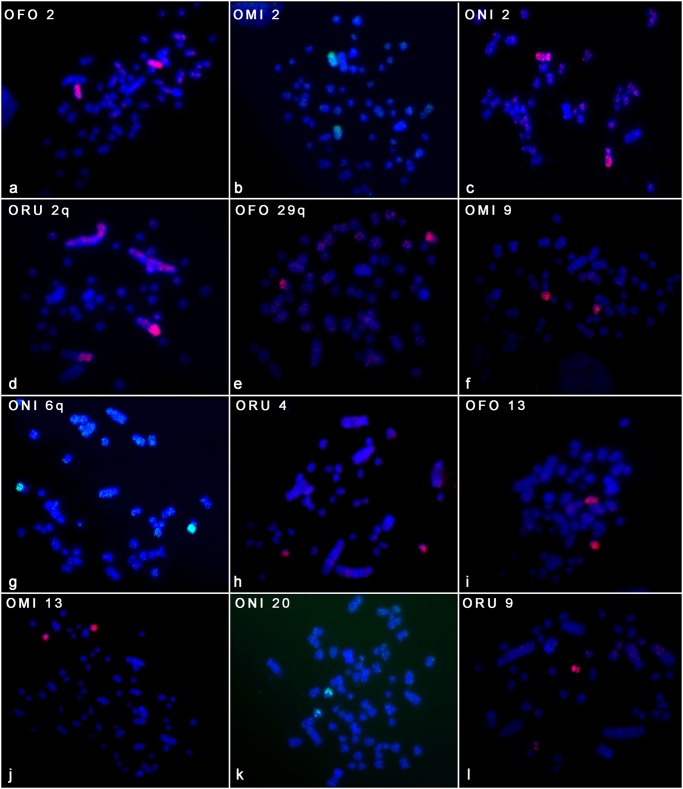
Compilation of hybridization patterns. **(a-d)** Hybridization using **OMO 2** paint in metaphases of *O*. *fornesi* (OFO), *O*. *microtis* (OMI), *O*. *nigripes* (ONI) and *O*. *rupestris* (ORU); **(e-h)** Hybridization using **OMO 8** paint in metaphases of *O*. *fornesi* (OFO), *O*. *microtis* (OMI), *O*. *nigripes* (ONI) and *O*. *rupestris* (ORU); **(i-l)** Hybridization using **OMO 17** paint in metaphases of *O*. *fornesi* (OFO), *O*. *microtis* (OMI), *O*. *nigripes* (ONI) and *O*. *rupestris* (ORU).

The 24 OMO probes were hybridized onto metaphases of *O*. *fornesi*, *O*. *microtis*, *O*. *nigripes*, *O*. *rupestris*, and *Oligoryzomys* sp. 2, the exception was *O*. *flavescens* in which only OMO Xa and OMO Xb were hybridized. The karyotypes of these species are shown in Fig. 2, including the pattern of hybridization found for each one. Also, a compilation of hybridization patterns is shown in Fig. 3.

### Chromosome painting with OMO Xa and Xb probes in *O*. *flavescens* (2n = 64+2Bs, FN = 66, XY)

*O*. *flavescens* has 2n = 64–66 and FN = 66, with 29 acrocentric pairs decreasing in size (1–29) and two small biarmed pairs (30 and 31). Pair 1 is the largest of the complement (Fig. 2b). Sex chromosome polymorphisms have been described as well as 1 to 4 supernumeraries (Bs) [14,17].

The hybridization of OMO Xa and OMO Xb probes onto metaphases of *O*. *flavescens*, male, with two Bs, showed signals in OFL X entirely and in the short arm of the OFL Y.

### Chromosome painting with *O*. *moojeni* probes in *O*. *fornesi* (2n = 62, FN = 64, XY)

The karyotype of *O*. *fornesi* comprises 28 acrocentric pairs (pair 1 being the largest of the complement), one medium submetacentric (pair 29) and one small metacentric (pair 30) (Fig. 2c) [24].

Cross-species hybridization of 24 OMO painting probes onto *O*. *fornesi* revealed 31 homologous segments (Table 2). Fourteen OMO paints (OMO 1, 2, 3, 7, 11, 12, 16, 17, 25, 26, 27, 28, 29 and 34) were hybridized to whole chromosomes of OFO (1, 2, 3, 8, 10, 11, 12, 13, 14, 15, 16, 24, 25 and 26, respectively). Five probes (OMO 4, 5, 6, 13 and 33) hybridized to more than one pair in *O*. *fornesi*. Thus, two pairs OFO 5 and OFO 29, showed associations with *O*. *moojeni* probes: OMO 4 + 5 and OMO 30 + 8, respectively. Finally, OMO Xa and Xb painted OFO X entirely and OFO Yp.

**Table 2:** Homologous segments detected by chromosome painting with *O*. *moojeni* (OMO) probes in metaphases of *O*. *fornesi* (OFO), *O*. *microtis* (OMI), *O*. *nigripes* (ONI), *O*. *rupestris* (ORU) and *Oligoryzomys* sp. 2 (OSP).

OMO Probes	OFO	OMI	ONI	ORU	OSP
(2n = 70)	(2n = 62)	(2n = 64)	(2n = 62)	(2n = 46)	(2n = 44)
OMO 1	1	1	128	3q	3q
OMO 2	2	2	2	2q	2q
OMO 3	3	3	3	1q	1q
OMO 4	5 (proximal)	5 (proximal)	7p	1q (3 interstitial segments)	1q (3 interstitial segments)
	17	17	13 (proximal)		
		26	17		
OMO 5	5 (distal)	5 (distal)	13 (distal)	1q (2 interstitial segments)	1q (2 interstitial segments)
	18	18	16		
OMO 6	22	22	4	2q	1p
	23	23	25	19	2q
				20	19
OMO 7	8	7	15	2q (interstitial)	2q (interstitial)
OMO 8	29q	9	6q	4	4
OMO 9,10	6 (distal)	4 (distal)	5p	3p	3p
	9	6	12 (distal)	12	12
			14	21	20
OMO 11	10	10	18	5	5
OMO 12	11	11	19	6	6
OMO 13	19	19	22	1q (interstitial)	1q (interstitial)
	20	20	23	13	13
	21	21	24	14	14
OMO 16	12	12	9q	1q	1q
OMO 17	13	13	21	9	9
OMO 25	14	14	20	1q	1q
OMO 26	15	15	26	1q	1q
OMO 27	16	16	27	2p	2p
OMO 28	24	24	29	3q	3q
				17	17
OMO 29	25	25	30	15	15
OMO 30	29p	27	6p	16	16
OMO 33	27	29	10	18	18
	28	30			
OMO 34	26	28	11	1q (interstitial)	1q (interstitial)
OMO Xa and Xb	X	X	X	X	X
	Yp	Yp	Yq		Yq
**Total**	**31 segments**	**32 segments**	**33 segments**	**33 segments**	**34 segments**

### Chromosome painting with *O*. *moojeni* probes in *O*. *microtis* (2n = 64, FN = 64, XY)

The karyotype of *O*. *microtis* exhibits 30 acrocentric pairs (pair 1 is the largest of the complement) decreasing in size and one small submetacentric (pair 31) (Fig. 2d) [14].

The hybridization of OMO probes onto OMI metaphases revealed 32 homologous segments (Table 2). Of the 24 probes, 16 (OMO 1, 2, 3, 7, 8, 11, 12, 16, 17, 25, 26, 27, 28, 29, 30 and 34) hybridized to whole chromosomes of OMI (OMI 1, 2, 3, 7, 9, 10, 11, 12, 13, 14, 15, 16, 24, 25, 27 and 28, respectively). Five paints (OMO 4, 5, 6, 13 and 33) hybridized to more than one pair in OMI. In addition, OMO 4 and OMO 5 were associated in pair OMI 5. Both the OMO Xa and Xb painted OMI X and OMI Yp.

### Chromosome painting with *O*. *moojeni* probes in *O*. *nigripes* (2n = 62, FN = 81, XY)

*O*. *nigripes* presents 2n = 62 with 11 biarmed pairs and 19 acrocentric pairs decreasing in size (Fig. 2e). The fundamental number of this species varies from 78 to 82 due to pericentric inversions in pairs 2, 3, 4 and 8, previously revealed by comparison of banding patterns [18].

Cross-species hybridization of OMO probes was performed onto *O*. *nigripes* with FN = 81, with pair 3 heteromorphic (metacentric/acrocentric) (Fig. 2e) and revealed 33 homologous segments between *O*. *moojeni* and *O*. *nigripes* (Table 2). Thirteen OMO paints (OMO 2, 3, 7, 11, 12, 17, 25, 26, 27, 28, 29, 33 and 34) hybridized to whole chromosomes of ONI (2, 3, 15, 18, 19, 21, 20, 26, 27, 29, 30, 10 and 11, respectively). Five paints hybridized to more than one autosomal pair (OMO 1, 4, 5, 6, 13) and three paints hybridized exclusively to autosomal arms (OMO 8, 16, 30). Two ONI pairs showed associations with OMO probes: ONI 6 (OMO 30 + 8) and ONI 13 (OMO 4 + 5).

OMO Xa and Xb probes both painted ONI X entirely and ONI Yq.

### Chromosome painting with *O*. *moojeni* probes in *O*. *rupestris* (2n = 46, FN = 52, XX)

The karyotype of *O*. *rupestris* is composed of macro- and micro-chromosomes, there being three large and 19 small pairs. Pair 1 is acrocentric, pairs 2 and 3 are subtelocentric and pairs 4 to 20 are acrocentrics graded by size. Also, pairs 21 and 22 are small metacentric and submetacentric, respectively (Fig. 2f) [15].

Cross-species chromosome painting with OMO probes onto ORU metaphases revealed 33 homologous segments (Table 2). OMO probes 8, 11, 12, 17, 29, 30 and 33 painted whole chromosomes of ORU (4, 5, 6, 9, 15, 16 and 18, respectively).

The three largest pairs of ORU showed associations with other OMO probes: pair ORU 1 was hybridized in 11 segments by eight probes (OMO 3, 4, 5, 13, 16, 25, 26 and 34); ORU 2 was hybridized by four different probes (OMO 2, 6, 7 and 27) and ORU 3 was hybridized by three probes (OMO 1, 9/10 and 28). OMO Xa and Xb painted ORU X entirely.

### Chromosome painting with *O*. *moojeni* probes in *Oligoryzomys* sp. 2 (2n = 44, FN = 52, XY)

The karyotype of *Oligoryzomys* sp. 2 is composed of three large and 18 small autosome pairs: 1, 2, and 3 are large subtelocentrics; pairs 4 to 19 are small acrocentrics graded in size, and pairs 20 and 21 are, respectively, small metacentric and submetacentric chromosomes. Fig. 2g shows only pair 1 and sex chromosomes of OSP2 since it is the only difference between OSP2 and ORU karyotypes.

The hybridization of OMO paints onto *Oligoryzomys* sp. 2 metaphases revealed 34 homologous segments (Table 2). Seven probes (OMO 8, 11, 12, 17, 29, 30 and 33) hybridized to whole chromosomes in OSP, showing a congruent pattern as observed in *O*. *rupestris*. Pairs OSP 2 and OSP 3 also showed a similar hybridization pattern as found in *O*. *rupestris*. However, nine probes painted OSP 1: OMO 3, 4, 5, 6, 13, 16, 25, 26 and 34.

In addition, OMO Xa and Xb painted OSP X and the pericentromeric region of OSP Y.

## Discussion

Zoo-FISH has been widely used for studies of chromosomal evolution, nevertheless this type of data is still scarce for Neotropical rodents of the subfamily Sigmodontinae.

The pioneering work with Zoo-FISH in Sigmodontinae rodents was performed by Fagundes et al. [25] with microdissected chromosome 1 of *Akodon cursor* (2n = 16). This probe was hybridized onto *A*. *cursor* (2n = 14 and 2n = 15) and *A*. *montensis* (2n = 24) metaphases and revealed several homologies.

Hass et al. [26] used *Mus musculus* probes to establish chromosome homology among *Akodon cursor*, *A*. *montensis*, *A*. *paranaensis* and *A*. *serrensis* and reconstructed the phylogenetic relationship among these species using *Oligoryzomys flavescens* as the outgroup.

Rodents of the tribe Akodontini were also studied by Ventura et al. [27] using reciprocal chromosome painting with *Akodon paranaensis*, *A*. *cursor* and *Akodon* sp. n. probes. This work revealed extensive chromosome rearrangements among *Akodon* species.

Up to now, the only study with species-specific paints in Oryzomyini tribe was performed by Nagamachi et al. [28] using *Hylaemys megacephalus* (2n = 54) probes in metaphases of *Cerradomys langguthi* (2n = 46). Results showed a large number of chromosome rearrangements. However, the authors did not consider the position of both species on the Oryzomyini phylogeny [5], so the rearrangements were merely descriptive with no correlation with the evolutionary context.

In the present paper, comparative chromosome painting, using *O*. *moojeni* probes in metaphases of five other *Oligoryzomys* species revealed extensive genomic reshuffling between very closely related species. Of the 24 *O*. *moojeni* probes, 14 (OMO 2, 3, 7, 8, 11, 12, 16, 17, 25, 26, 27, 29, 30 and 34) produced single signals on the karyotype of the five species studied (*O*. *fornesi*, *O*. *microtis*, *O*. *nigripes*, *O*. *rupestris*, and *Oligoryzomys* sp. 2), showing that the homology of these chromosomes is conserved (some examples are shown in Fig. 3). The remainder of the probes produced more than one hybridization signal in at least one species, showing that they are rearranged in the genomes of the species studied.

Independently of the use of a phylogeny to interpret the direction of the rearrangements, is it possible to infer by our hybridization results that extensive chromosomal rearrangements such as pericentric inversions or repositioning of centromeres, Robertsonian rearrangements and tandem fusion/ fission, gain/ activation or loss/inactivation of centromeres and telomeric sequences have occurred in these closely related species of *Oligoryzomys*.

**Fig 4:**
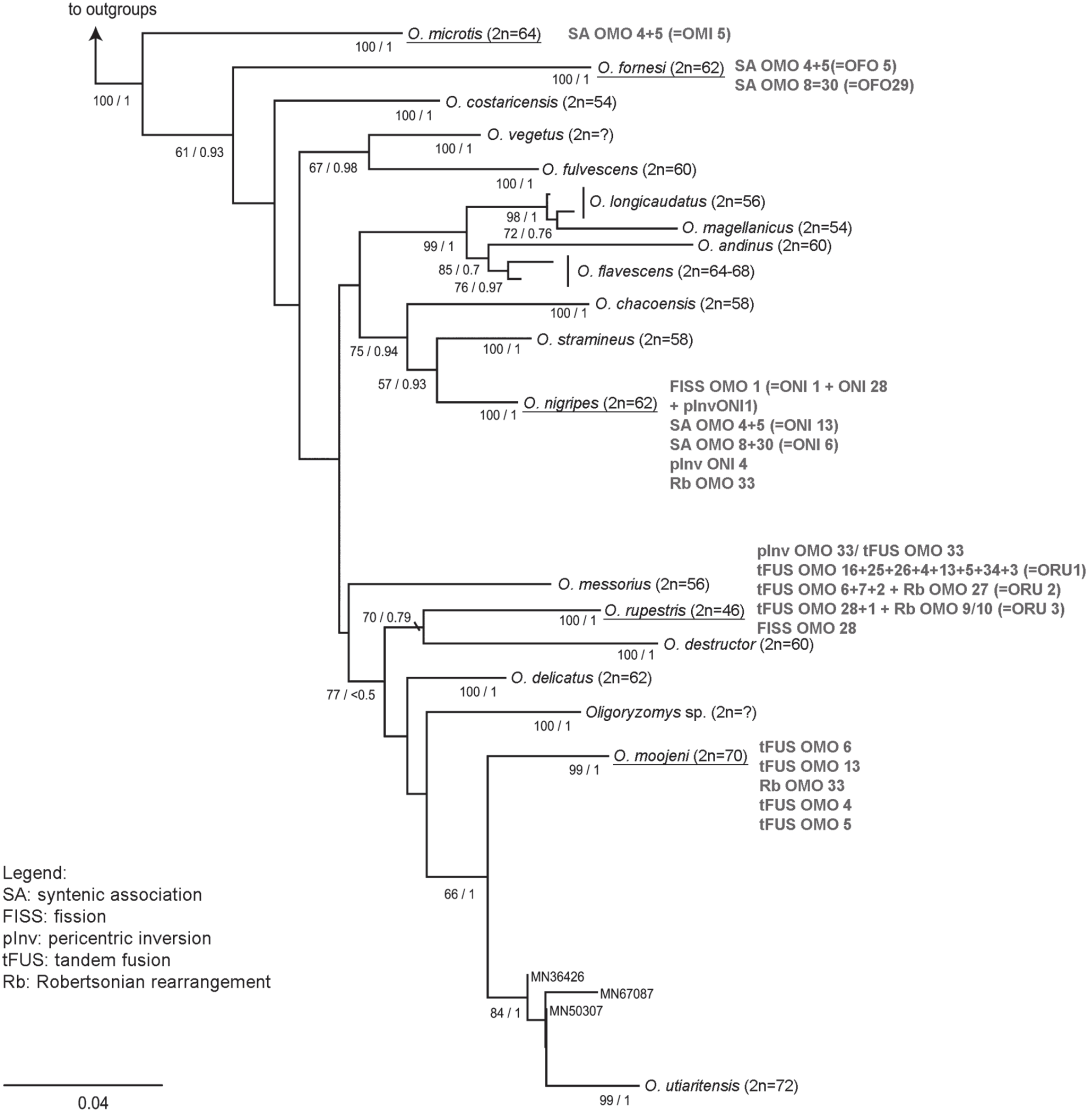
Phylogenetic relationships of *Oligoryzomys*. Phylogenetic relationships of *Oligoryzomys* based on cytochrome b gene (extracted and modified from Agrellos et al. [12]). Outgroup taxa included *Oreoryzomys balneator*, *Microryzomys minutus*, *Oryzomys palustris* and *Neacomys spinosus*. Nodal support values shown above the branches are ML bootstrap values (>50%) and Bayesian posterior probability (> 0.50). *Oligoryzomys* sp. 2 is not represented in the tree. The main rearrangements detected are plotted next to the name of each species studied herein (underlined).

Additionally, in order to infer the chromosome rearrangements directions that have occurred during the karyotype evolution of the genus, we compared our results of chromosome painting to the phylogeny of *Oligoryzomys* based on molecular data, published by Agrellos et al. [12], in which we highlighted the rearrangements obtained herein (Fig. 4, Table 2). This molecular phylogeny encompasses the highest number of species, including all the species studied herein, except for *Oligoryzomys* sp. 2 that represent an undescribed species.

### Chromosomal rearrangements in *Oligoryzomys* revealed by chromosome painting

Comparative G-banding patterns have indicated total homology among the largest chromosome (pair 1) of five *Oligoryzomys* species [14]. However, in *O*. *nigripes*, cross-species chromosome painting with OMO 1 showed that this chromosome is homologous to two chromosomes pairs, ONI 1 and 28 (Table 2). Considering the current phylogeny, we can infer the occurrence of fission involving ONI 1 and ONI 28, followed by pericentric inversion or centromere repositioning events yielding the subtelocentric ONI 1.

OMO 6 painted two acrocentric pairs in *O*. *fornesi* (OFO 22 and OFO 23) and *O*. *microtis* (OMI 22 and OMI 23), indicating that tandem fusion occurred in *O*. *moojeni*. In *O*. *nigripes*, the same probe painted one acrocentric (ONI 25) and one metacentric pair (ONI 4), showing that pericentric inversion also occurred in this species.

Concerning OMO 13, this probe hybridized to three pairs in OFO, OMI and ONI, showing that at least two events of tandem fusion have occurred in *O*. *moojeni*.

OMO 28, that is conserved in *O*. *fornesi*, *O*. *microtis* and *O*. *nigripes* is disrupted in *O*. *rupestris* and *Oligoryzomys* sp. 2 (ORU/ OSP 3q and 17), showing that fission rearrangement has occurred even in the species with the lowest diploid number.

OMO 33 is a small metacentric chromosome that is conserved in *O*. *nigripes*. Nevertheless, in *O*. *fornesi* and *O*. *microtis*, this probe painted two small acrocentric pairs, revealing the occurrence of a centric fusion rearrangement in ONI and OMO. Also, in *O*. *rupestris* and *Oligoryzomys* sp. 2, the same probe painted one acrocentric pair, showing that pericentric inversion or tandem fusion events should have occurred in these two species.

### Specific associations revealed by chromosome painting

**Fig 5:**
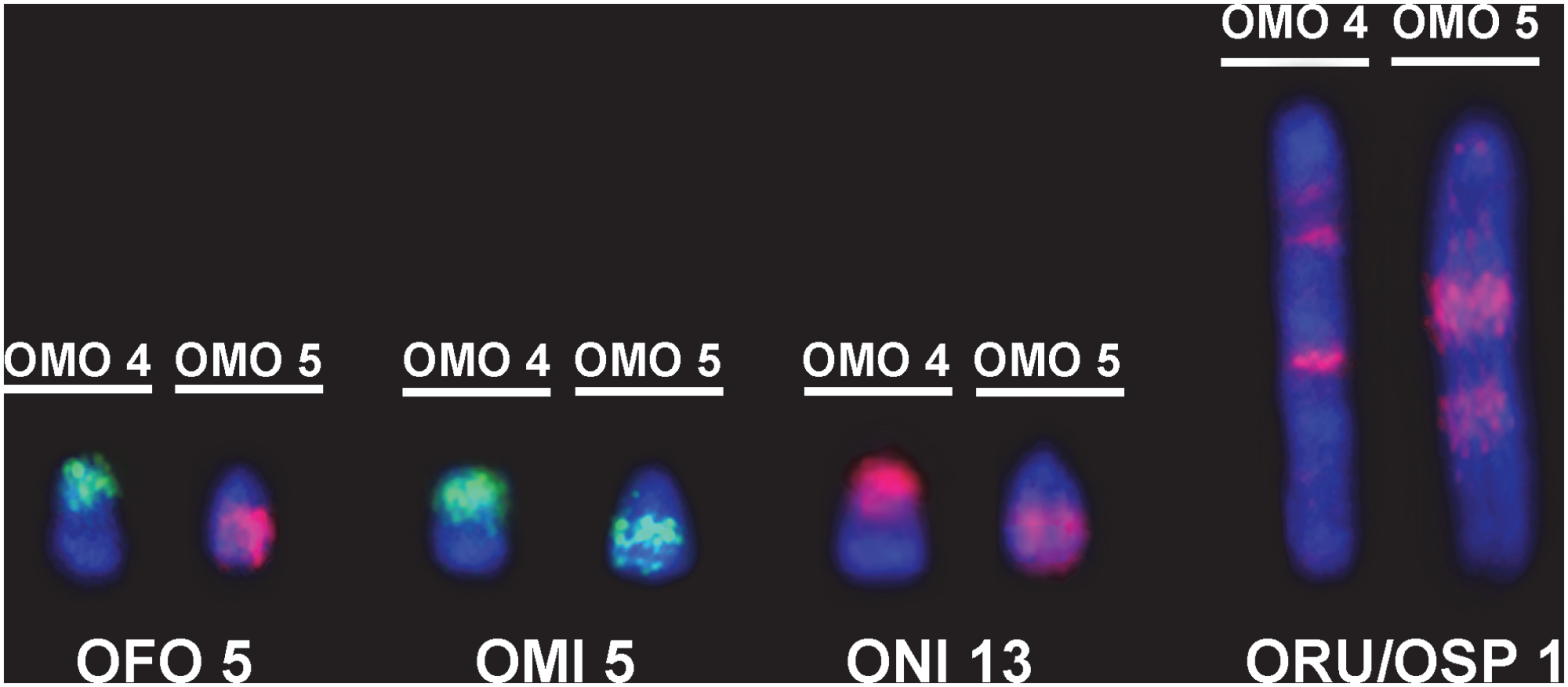
Specific associations of probes OMO 4 and OMO 5. *O*. *fornesi* (OFO), *O*. *microtis* (OMI), *O*. *nigripes* (ONI), *O*. *rupestris* (ORU) and *Oligoryzomys* sp. 2 (OSP), respectively.

Cross-species chromosome painting showed that probes OMO 4 and OMO 5 are associated in *O*. *fornesi* (OFO 5), *O*. *microtis* (OMI 5) and *O*. *nigripes* (ONI 13). Both probes also hybridized in the same pair in *O*. *rupestris* (ORU 1q) and *Oligoryzomys* sp. 2 (OSP 1q) (Fig. 5). Additionally, as showed in Table 2, OMO 4 painted a total of three segments in *O*. *microtis*, *O*. *nigripes*, *O*. *rupestris* and *Oligoryzomys* sp. 2, and two segments in *O*. *fornesi* while OMO 5 painted two segments in all species, showing that complex rearrangements are involved in the karyotype evolution of the different species regarding these chromosomes.

**Fig 6:**
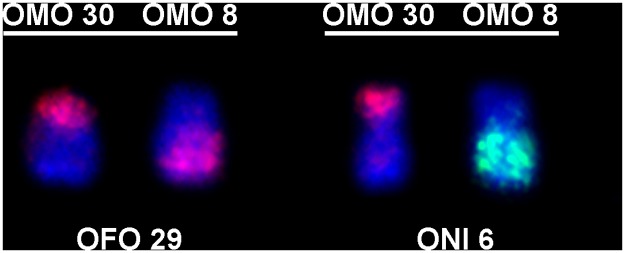
Specific associations of probes OMO 30 and OMO 8. *O*. *fornesi* (submetacentric OFO 29) and *O*. *nigripes* (submetacentric ONI 6), respectively.

Besides, probes OMO 30 and OMO 8 were also associated in *O*. *fornesi* (OFO 29) and *O*. *nigripes* (ONI 6) (Fig. 6). According to the phylogeny, this syntenic association could have emerged independently in the lineages of both species or could have appeared in the common ancestor of *O*. *fornesi* and *O*. *nigripes* and reversed in the common ancestor of *O*. *rupestris* and *O*. *moojeni*.

### The reduction of diploid number in *O*. *rupestris* and *Oligoryzomys* sp. 2

**Fig 7:**
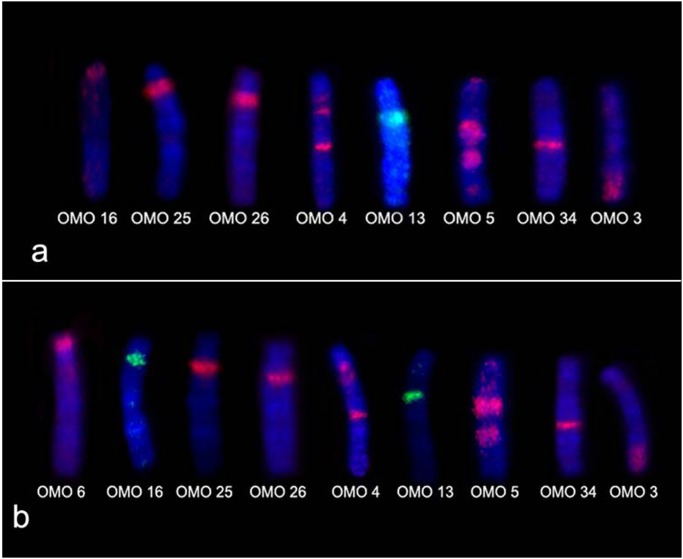
Tandem fusions in pair 1 of low diploid number species. **(a)** *O*. *rupestris* (2n = 46) chromosome 1 hybridized by eight different *O*. *moojeni* probes; **(b)** *Oligoryzomys* sp. 2 (2n = 44) chromosome 1 hybridized by nine different *O*. *moojeni* probes.

**Fig 8:**
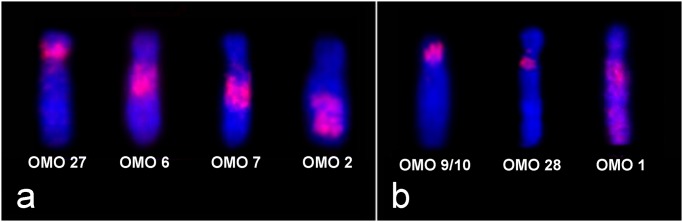
Tandem fusions in pairs 2 and 3 of low diploid number species. **(a)** *O*. *rupestris* and *Oligoryzomys* sp. 2 chromosome 2 hybridized by four different *O*. *moojeni* probes; **(b)** *O*. *rupestris* and *Oligoryzomys* sp. 2 chromosome 3 hybridized by three different *O*. *moojeni* probes.

Chromosome painting in *O*. *rupestris* (2n = 46) and *Oligoryzomys* sp. 2 (2n = 44) showed almost the same hybridization pattern. As *O*. *rupestris* and *Oligoryzomys* sp. 2 have very similar karyotypes and totally different from other species of the same genus, it is likely that they are closely phylogenetically related. In *O*. *rupestris*, pair 1 was hybridized by eight OMO probes while nine probes hybridized pair 1 of *Oligoryzomys* sp. 2 (Fig. 7). In addition, ORU 2/ OSP 2 were hybridized by four probes and ORU 3/ OSP 3 by three probes (Fig. 8), showing that tandem fusion events occurred during karyotype differentiation of both species, providing the lowest known diploid number of the genus.

We also should bring here again the information regarding OMO 4 and OMO 5 probes, which are disrupted in all species studied and this situation cannot be showed clearly in Fig. 4 (see table 2).

Results indicate loss, inactivation or centromeric repositioning, since tandem fusions formed the three largest pairs of *O*. *rupestris* and *Oligoryzomys* sp. 2. In addition, as we were not able to use all probes, more chromosomes than observed here can be involved in tandem fusions in these pairs.

**Fig 9:**
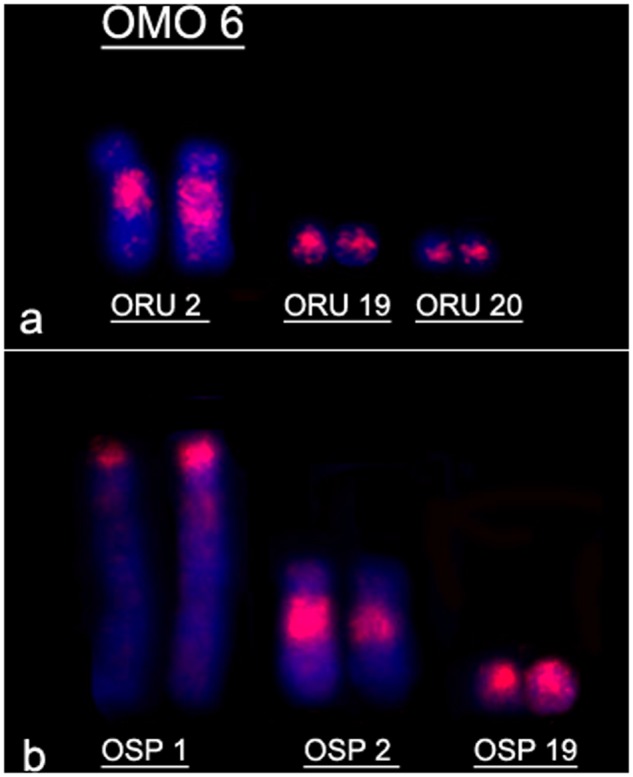
Hybridization pattern of probe OMO 6 showing a centric fusion rearrangement. **(a)** *O*. *rupestris* (2n = 46); **(b)** *Oligoryzomys* sp. 2 (2n = 44).

Chromosome painting corroborates previous data that centric fusion was the mechanism responsible for the difference in diploid number of both species [15] since probe OMO 6 painted three segments in *O*. *rupestris* (ORU 2q, ORU 19 and ORU 20) and *Oligoryzomys* sp. 2 (OSP 1p, OSP 2q and OSP 19) (Fig. 9).

### Sex chromosomes homologies

Both OMO Xa and OMO Xb hybridized to the whole X chromosome of all species, corroborating that the mammalian X chromosome is conserved.

In *O*. *flavescens*, OMO Xa and Xb did not show positive signals in supernumerary chromosomes, indicating that in the case of this species, no homology were found between Bs and X chromosome. In contrast, Silva and Yonenaga-Yassuda [29] showed homology of *Nectomys squamipes* B chromosome and the constitutive heterochromatin the X chromosome of this species.

**Fig 10:**
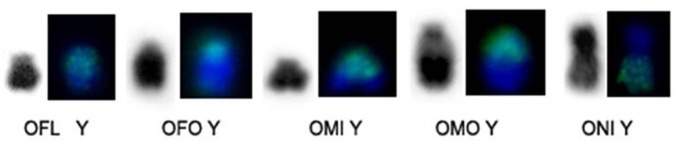
Hybridization pattern of OMO X chromosome. OMO Xa and OMO Xb hybridized in euchromatic region of Y chromosome of *O*. *flavescens*, *O*. *fornesi*, *O*. *microtis*, *O*. *moojeni* and *O*. *nigripes*, respectively.

In *O*. *flavescens*, *O*. *fornesi*, *O*. *microtis*, *O*. *moojeni*, *O*. *nigripes* and *Oligoryzomys* sp. 2, OMO Xa and OMO Xb showed homology with the euchromatic region of Y chromosomes (Fig. 10). This could be related to the pseudoautosomal region as described in other rodents of the subfamily Arvicolinae and Sigmodontinae as well [30–32].

### Chromosome evolution in *Oligoryzomys*

The present study shows that complex rearrangements are involved in the karyotype evolution of *Oligoryzomys* species. Nevertheless, FISH data using telomeric probes demonstrate signals exclusively at the telomeres of *Oligoryzomys* sp. 2 and *O*. *rupestris* [15] and in the remaining species studied here: *O*. *flavecens*, *O*. *fornesi*, *O*. *microtis*, *O*. *moojeni* and *O*. *nigripes* [14]. One possible explanation is that telomeric repeats may have been eliminated by chromosome breakages [33]. On the other hand, the presence of interstitial telomeric sequences (ITS) could not mean that these sequences are involved in rearrangements, since ITS were also observed in highly conserved karyotypes [34] and blocks of constitutive heterochromatin [35,36].

There are relatively few studies that compare chromosome painting with a phylogeny based on molecular data. According to the phylogeny, we could infer the occurrence of many tandem fusion events, since single OMO probes (the most derived species of the genus) painted two or more regions of different chromosomes of *Oligoryzomys microtis* (that have diverged earlier in the phylogeny) (Table 2).

However, the phylogenetic relationships of *Oligoryzomys* species remain unclear and this is the first phylogeny in which *O*. *moojeni* belongs to the most derived clade [12]. Still, we consider it important to integrate cytogenetic and molecular data in order to understand the karyotype evolution of such a complex genus as *Oligoryzomys*.

## Conclusions

Chromosomal evolution data in *Oligoryzomys* is being presented for the first time. Cross species chromosome painting revealed an extensive chromosomal reorganization despite the absence of interstitial telomeric sequence. *Oligoryzomys rupestris* and *Oligoryzomys* sp. 2 are the species with the most rearranged karyotypes and at least 17 tandem fusions originate the three largerst pairs of both species. Besides, a centric fusion originates the difference in diploid number of both species (2n = 46 and 2n = 44, respectively). Chromosome painting in *Oligoryzomys* species shows that pericentric inversions, fissions, tandem and Robertsonian fusions, centromeric loss/ inactivation, gain/activation or repositioning have occurred during karyotype evolution of the genus.

Also, although it is not easy to determine the direction of chromosome change, it is possible to infer that in *Oligoryzomys*, chromosomal evolution has been associated with both, decrease and increase in diploid numbers, in contrast with other groups in which diploid number tends to decrease during their evolution. These data indicate that all those closely related species have experienced recent autosomal rearrangement.
